# Effects of Low Protein-High Carbohydrate Diet during Early and Late Pregnancy on Respiratory Quotient and Visceral Adiposity

**DOI:** 10.1155/2022/3878581

**Published:** 2022-04-07

**Authors:** Mónica Navarro-Meza, Mauricio Díaz-Muñoz, Perla Belén García-Solano, Raquel Cobián-Cervantes, Éricka A. de los Ríos-Arellano, Felipe Santoyo Telles, Mariela Camacho-Barrón

**Affiliations:** ^1^Centro Universitario del Sur, Laboratory Clinic of Neuronutrition and Memory, Departamento de Promoción, Preservación y Desarrollo de la Salud, Universidad de Guadalajara, Ciudad Guzmán, Jalisco, 49000, Mexico; ^2^Departamento de Neurobiología Celular y Molecular, Instituto de Neurobiología, Universidad Nacional Autónoma de México, Querétaro, 76230 QRO, Mexico; ^3^Centro Universitario del Sur, Universidad de Guadalajara, Ciudad Guzmán, Jalisco, 49000, Mexico; ^4^Facultad de Ciencias Naturales, Universidad Autónoma de Querétaro, Av de las Ciencias S/N, Juriquilla, 76230 Querétaro, Mexico

## Abstract

**Background:**

Low Protein-High Carbohydrate (LPHC) diet during pregnancy is considered a nutritional and health problem related to the development of maternal metabolic alterations, such as fatty liver and obesity in the perinatal and postnatal period. It is known that increase in visceral adiposity tissue (VAT) modulates maternal metabolic rate, with the respiratory quotient (RQ) being a parameter related to that variable; however, it is unknown whether LPHC intake during pregnancy affects the VAT and the RQ. In this study, we examine if consumption of LPHC during pregnancy modifies the VAT and RQ in early and late periods of pregnancy.

**Methods:**

This is a longitudinal and cross-sectional study with Wistar rats during gestation (G) (3, 8, 15, and 20) and nonpregnant rats. Rats were fed with a control diet with 63/18% carbohydrate/protein and an experimental diet with 79/6% carbohydrate/protein. We studied water and food consumption and metabolic parameters such as RQ and energy expenditure (EE), calculated by indirect calorimetry. In the cross-sectional study, we determined visceral fat, as well as the concentration of free fatty acids, insulin, glucose, and lipid profile in serum.

**Results:**

Nonpregnant rats with LPHC intake decreased significantly in VAT (86%) and the RQ (18%); in pregnant rats in early (8G) and late pregnancy (15G) in LPHC diet, both parameters (VAT and RQ) (25%-92%) increased during light time. When comparing time points during pregnancy in the control and LPHC groups, the RQ increased in 15G during daytime compared to 8G during the night period (17 and 5%, respectively). In late pregnancy, LPHC intake and triacylglyceride levels increased and cholesterol and glucose decreased (45 and 26%, respectively), in comparison to nonpregnant rats.

**Conclusions:**

LPHC intake in nonpregnant rats decreases the RQ and VAT. Interestingly, the opposite occurs in early pregnancy: the RQ and VAT increased, and this correlates with free fatty acid (FFA) levels. The increase in RQ and VAT during light time in early pregnancy increased mobilization of carbohydrate and protein metabolism. These results suggest that LPHC intake during pregnancy increases the glucose metabolism as a compensatory mechanism for energy needs in the fetus and the mother in early pregnancy.

## 1. Introduction

A nutritionally deficient diet with LPHC before and during pregnancy is associated with the development of metabolic disorders, as well as with perinatal complications [1]. The consumption of a LPHC during pregnancy increases visceral fat tissue (VAT) with the consequent detrimental maternal and fetal health [2]. It promotes newborn difficulties such as fetal macrosomia, metabolic disorders, hypoglycemia, and respiratory distress. Studies have shown that the distribution of adipose tissue is an important predictor of morbidity and mortality even better than the body mass index [3]. During pregnancy, increased VAT can affect maternal health and fetal growth and favor physiological responses in the fetus that predispose the offspring to metabolic and cardiovascular disease [4, 5]. Few studies have investigated how adipose tissue distribution changes during pregnancy in relation to the consumption of different types of diets [6]. The increase in VAT disturbs negatively reproductive health and influences childbirth and the puerperium, with a greater number of maternal-fetal complications. In this regard, more than 20% of pregnancy difficulties are promoted by maternal alterations in adiposity [7].

It is known that during pregnancy, the maternal basal metabolic rate increases both the resting energy expenditure (EE) and the total energy expenditure, in order to support the growth and development of the offspring [8]. It has been suggested that increased gestational weight gain and accumulation of fat mass are inversely related to changes in resting EE [9]. The respiratory quotient (RQ) is a metabolic indicator based on the ratio of the volume of carbon dioxide produced and the oxygen consumed [10]. RQ is a parameter that also indicates the type of nutrient that is being oxidized, either carbohydrates (RQ 1), lipids (RQ 0.7), proteins (0.8), or a mixture [11]. During pregnancy, there is an adaptation of the intermediary metabolism to meet fetal energy demands and placental development. In the early stage of pregnancy, under normal conditions, the glucose and fatty acid uptake is facilitated by adipose tissue, producing an increase in fat reserves. However, as pregnancy progresses, there is a change in the metabolism characterized by insulin resistance and a decrease in glucose metabolism [12]. In pregnant women with normal weight, the RQ is not significantly modified, although they show lower values in conditions of overweight and obesity [3]. Given that the increase in body weight and adiposity during pregnancy is mainly regulated by the imbalance between energy intake and RQ, this research raises the question: does a LPHC intake during early and late pregnancy affect RQ and development of VAT as metabolic variables?

## 2. Methods

The procedures were carried out in accordance with the Official Mexican Standard NOM-062-ZOO-1999.

This research was reviewed and authorized by the Ethics Committee of the Neurobiology Institute, UNAM (México).

### 2.1. Experimental Longitudinal and Transversal Study

The animals were individually held in cages (21 × 23.5 × 38 cm) under controlled 12 h dark/light conditions with lights on at 07:00 h. A progressive study was done in mothers during gestation (G) days (3, 8, 15, and 20). In this study, we classified early pregnancy at gestational day 3 (G) and 8G and late pregnancy at 15G and 20G. *Wistar* rats, weighing 250-300 g, were randomly divided into 4 groups:

1. Control diet nonpregnant (*n* = 4)
2. LPHC diet nonpregnant (*n* = 4)
3. Control diet pregnant (CP): early pregnancy (*n* = 3, 3G; 8G) and late pregnancy (*n* = 3, 15G; 20G)
4. LPHC diet pregnant: early pregnancy (*n* = 3, 3G; 8G) and late pregnancy (*n* = 3, 15G; 20G)

Before pregnancy and during pregnancy, food and water intake was quantified, as well as body weight using a precision scale (A&D Weighing series GF-3000 scale, South Korea).

A control diet (AIN 93G Test diet, 63/18%) (63.2% carbohydrate, 7.1% fat, and 18% protein) and experimental diet (LPHC) (AIN 93G Test diet, 77/6%) (77.3% carbohydrate, 7.1% fat, and 6% protein) were used. All groups had access to diets *ad libitum*. On days 3, 8, 15, and 20 of pregnancy, blood samples were obtained and fat tissue was dissected. Blood was collected in Eppendorf tubes, followed by centrifugation at 400g for 10 min to obtain serum, which was stored in aliquots at −20°C until the analysis.

We used 48 female Wistar rats from 250 to 400 g, aged 4 to 5 months: 14 for the longitudinal study and 34 for the transversal study. They were placed in metabolic cages to record the energy expenditure (EE), respiration quotient (RQ), and food and water consumption per day. The 12 h light and dark exposure cycle was maintained with the lights on at 07:00 h.

The rats had free access to water and the experimental diet (LPHC, isocaloric high in carbohydrates and low in protein). The proestrus-estrus stage was identified by a vaginal smear. Once this stage was recorded, each female rat was placed with a male rat until 3 ejaculations were accomplished in order to ensure fertilization (gestational day 0, G0; the vaginal smear was also checked). A precision electronic scale A&D Weighing Series GF3000 was used to record the body weight. In parallel, the control diet was used; the two study groups consumed the food *ad libitum* during the pregnancy stage. They were sacrificed according to the current standard of care and handling of experimental animals. The respirometry studies were longitudinal, while those of blood and adiposity markers were cross-sectional.

### 2.2. Diet, Food and Water Intake, and Body Weight

The longitudinal experiment was carried out in the metabolic cages on days 8 and 15 of gestation, and nonpregnant females were exposed to the LPHC and the control diet. The cross-sectional study included sacrifice by decapitation on gestational days 8 and 15 with the aim of obtaining visceral fat and peripheral blood from pregnant mothers and nonpregnant females to determine biochemical parameters.

### 2.3. Evaluation of Indirect Calorimetry

The amount of food and water consumed every 24 h was individually recorded in metabolic cages (Oxylet LE 1305 Physiocage, Panlab, Panlab Harvard, Barcelona, Spain). For this purpose, female rats were placed in individual acrylic cages in temperature-controlled environment (23 ± 2°C) with a 12 h : 12 h photoperiod (lights on at 07:00 h). Rats were acclimatized in the cages for 24 h before experimental measurements. Oxygen consumption (VO_2_) and carbon dioxide production (VCO_2_) were measured every 12 min during 24 h using an O_2_ and CO_2_ analyzer (Oxylet LE 405-gas analyzer, Panlab) at a controlled flow rate of 900 ml/min (Oxylet LE 400-air supplier, Panlab). At each point, we used the software v3.0.01 metabolism (Panlab Harvard Apparatus, Barcelona, Spain) for the quantification of respiratory quotient (RQ) based on the VCO_2_/VO_2_ ratio and energy expenditure (EE) in kcal/day. During all procedures, rats had free access to control and experimental diets and water *ad libitum*. Locomotor activity as well as food and water intake was measured by continuous recording using the Panlab weight transducer (LE1305 sensor platform, Panlab).

### 2.4. Adiposity Analysis

The VAT was obtained from both pregnant and nonpregnant rats that consumed the control diet and the LPHC; the ratio of adipose tissue to body weight was also obtained, on days 8 and 15 of gestation. Finally, nonpregnant female rats were exposed to the experimental and control diet; VAT was obtained from the abdominal region in nonpregnancy, early pregnancy, and late pregnancy.

### 2.5. Serum Free Fatty Acid Levels

Free fatty acid levels were determined with a reagent kit (MAK 044) from Sigma-Aldrich. All procedures were followed according to the manufacturer's instructions.

### 2.6. Biochemical Determinations

Blood samples were obtained by rapid decapitation, in order to obtain serum to determine glucose and insulin levels and the lipid profile. Spinreact kits from Ctra. (Santa Coloma, Spain) were used to determine the levels of total cholesterol, triacylglycerides, high-density lipoproteins, and glucose.

### 2.7. Statistical Analysis

Statistical analyses were performed with SPSS software (version 19), considering a value of *p* < 0.05 to be statistically significant. The Kolmogorov-Smirnov and the Shapiro-Wilk test were used to test the normality of the biochemical variables. To compare the control diet group with the LPHC group, Student's *t*-test, mediated by Levene's test, was used. A one-way ANOVA was used to compare the early and late pregnant rats, control, and LPHC groups. Two-way ANOVA was used to relate the two categorical variables with one interval.

## 3. Results

**Figure 1:**
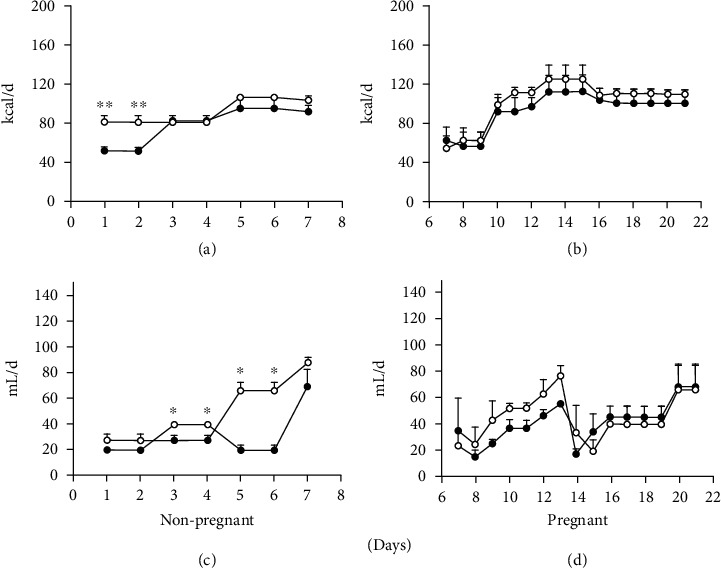
Food and water intake and body weight during nonpregnancy and pregnancy: (a) food intake in nonpregnant rats (kcal/d); (b) food intake in pregnant rats (kcal/d); (c) water intake in nonpregnant rats (ml/d); (d) water intake in pregnant rats (ml/d). Data represent the mean and standard error of three independent experiments (^∗^*p* < 0.05). The black circles represent the group of rats with control diet while the white circles correspond to the group of rats with low-protein diet.

**Figure 2:**
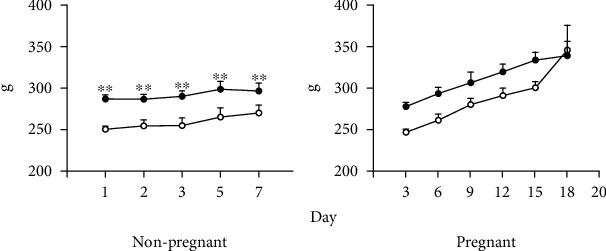
Weight gain in nonpregnant and pregnant rats: (a) weight gain in nonpregnant rats (g/d) and (b) weight gain in pregnant rats (g/d). Data represent the mean and standard error of three independent experiments (^∗^*p* < 0.05). The black circles represent the group of rats with control diet while the white circles correspond to the group of rats with low-protein diet.

**Figure 3:**
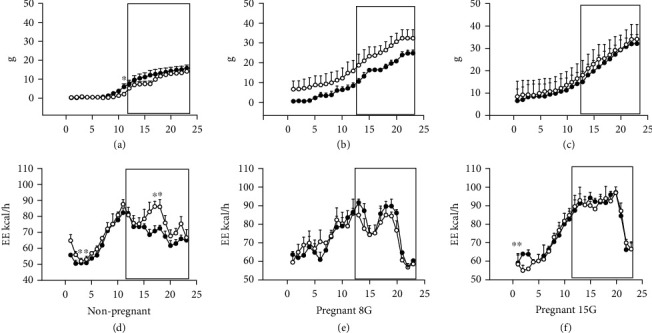
Food intake and energy expenditure in nonpregnant and pregnant rats on gestation days 8 and 15 (early pregnant (8G) and late pregnant (15G)) during 24 h. Data were collected using metabolic cages (Panlab, Spain). The section inside the box represents the dark phase (the light-off); the black circles represent the group of rats with control diet while the white circles correspond to the group of rats with low-protein diet: (a) food intake, expressed in grams (g), during 24 h in nonpregnant rats; (b) food intake, expressed in g, during 24 h in early pregnancy rats in 8 days of gestation (8G); (c) food intake, expressed in g, during 24 h in late pregnancy rats in 15 days of gestation (15 G); (d) energy expenditure recorded for 1 day, in nonpregnant rats; (e) energy expenditure recorded for 1 day, in pregnant rats in 8G; (f) energy expenditure recorded for 1 day in pregnant rats in 15G. Data represent the mean and standard error of three independent experiments (^∗^*p* < 0.05).

The results are described below; first, we indicate the results related to food and water intake and body weight in the control and the experimental groups (rats that consumed a low-protein diet (LPHC); nonpregnant rats and pregnant rats were examined on day 8 (early pregnancy) and day 15 of gestation (late pregnancy) (Figures 1 and 2). Subsequently, we show the results obtained in metabolic boxes (food consumption and energy expenditure) in nonpregnant rats and pregnant rats at day 8 (early pregnancy) and day 15 of gestation (late pregnancy) (Figures 3(a)–3(f)).

**Figure 4:**
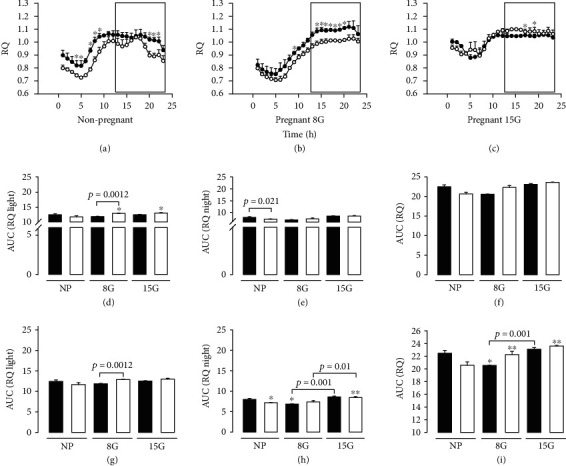
Respiratory quotient (ratio of CO_2_ produced by the O_2_ consumed) recorded for 24 h in nonpregnant rats and pregnant rats. Data were collected using metabolic cages (Panlab, Spain). The section inside the box represents the dark phase (the light-off); the black circles represent the group of rats with control diet while the white circles correspond to the group of rats with low-protein diet; the black rectangles correspond to rats with a control diet, and white rectangles indicate rats with a low-protein diet. AUC: arbitrary units of the area under the curve. (a) Respiratory quotient (ratio of CO_2_ produced by the O_2_ consumed) recorded for 24 h in nonpregnant rats (NP), (b) respiratory quotient (ratio of CO_2_ produced by the O_2_ consumed) recorded for 24 h in pregnant rats (8G), and (c) respiratory quotient (ratio of CO_2_ produced by the O_2_ consumed) recorded for 24 h in pregnant rats (15G). (d) The black bars (rectangles) correspond to the group of rats with control diet and indicate the area under the curve of respiration coefficient during the light period. NP: nonpregnant; 8G: gestation day 8; 15G: gestation day 15. The white bars correspond to the group of rats that consumed the low-protein diet and represent the area under the curve of the respiration coefficient during the light period. NP: nonpregnant; 8G: gestation day 8; 15G: gestation day 15 (intergroup comparisons). (e) The black bars (rectangles) correspond to the group of rats that consumed the control diet and represent the area under the curve of the respiration coefficient during the night period. NP: nonpregnant; 8G: gestation day 8; 15G: gestation day 15. The white bars correspond to the group of rats that consumed the low-protein diet and represent the area under the curve of the respiration coefficient during the light period. NP: nonpregnant; 8G: gestation day 8; 15G: gestation day 15 (intergroup comparisons). (f) The black bars (rectangles) correspond to the group of rats that consumed the control diet and represent the area under the curve of the respiration coefficient during 24 h. NP: nonpregnant; 8G: gestation day 8; 15G: gestation day 15. The white bars correspond to the group of rats that consumed the low-protein diet and represent the area under the curve of the respiration coefficient during the light period. NP: nonpregnant; 8G: gestation day 8; 15G: gestation day 15 (intergroup comparisons). (g) The black bars correspond to the group of rats that consumed the control diet and represent the area under the curve of the respiration coefficient during the light period. NE: nonpregnant; 8G: gestation day 8; 15G: gestation day 15. The white bars correspond to the group of rats that consumed the low-protein diet and represent the area under the curve of the respiration coefficient during the light period. NP: nonpregnant; 8G: gestation day 8; 15G: gestation day 15 (intragroup comparisons). (h) The black bars correspond to the group of rats that consumed the control diet and represent the area under the curve of the respiration coefficient during the night period. NP: nonpregnant; 8G: gestation day 8; 15G: gestation day 15. The white bars correspond to the group of rats that consumed the low-protein diet and represent the area under the curve of the respiration coefficient during the light period. NE: nonpregnant; 8G: gestation day 8; 15G: gestation day 15 (intragroup comparisons). (i) The black bars correspond to the group of rats that consumed the control diet and represent the area under the curve of the respiration coefficient during 24 h. NP: nonpregnant; 8G: gestation day 8; 15G: gestation day 15. The white bars correspond to the group of rats that consumed the low-protein diet and represent the area under the curve of the respiration coefficient during the light period. NP: nonpregnant; 8G: gestation day 8; 15G: gestation day 15 (intragroup comparisons). Data represent the mean and standard error of three independent experiments (^∗^*p* < 0.05).

**Figure 5:**
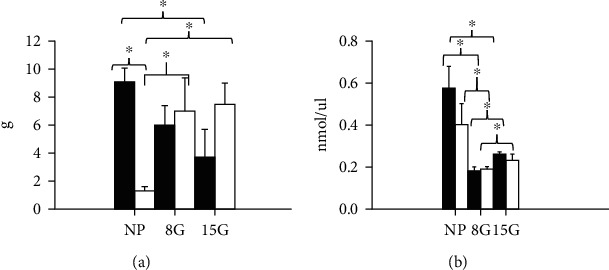
Visceral adipose tissue and serum free fatty acid levels in nonpregnancy and pregnancy on the 8th day of gestation (early pregnancy) and on the 15th day of gestation (late pregnancy). (a) Visceral adipose tissue/body weight ratio in not pregnant (NP), early pregnancy (8G), and late pregnancy (15G) rats and (b) free fatty acid in serum in nonpregnant (NP), early pregnancy (8G), and late pregnancy (15G) rats. Data represent the mean and standard error of three independent experiments (^∗^*p* < 0.05).

**Figure 6:**
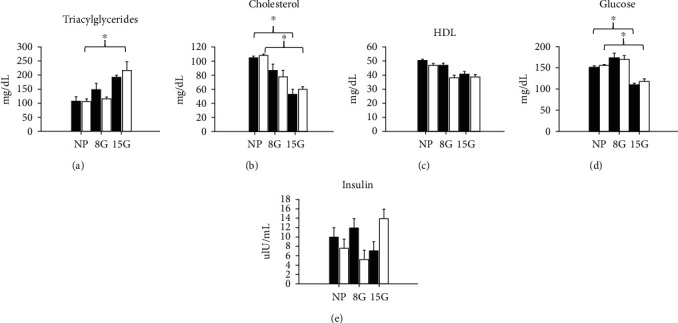
Biochemical markers in serum. The black bars correspond to nonpregnant (NP) and pregnant rats on gestation day 8 (8G) and gestation day 15 (15G) with control diet, and white bars indicate nonpregnant and pregnant rats on 8G and 15G with low-protein diet (LPHC). It showed (a) triacylglycerides, (b) cholesterol, (c) HDL, (d) glucose, and (e) insulin. Data represent the mean and standard error of three independent experiments (^∗^*p* < 0.05).

In Figure 4, we show the RQ and the area under the curve in nonpregnant rats and pregnant rats on day 8 (early pregnancy) and day 15 gestation (late pregnancy) (Figures 4(d)–4(i)). In Figure 5, we show visceral fat in nonpregnant rats and pregnant rats at day 8 (early pregnancy) and day 15 of gestation (late pregnancy) and free fatty acids. In addition, in Figure 6, we indicate the serum markers in nonpregnant rats and pregnant rats at day 8 (early pregnancy) and day 15 of gestation (late pregnancy).

### 3.1. Food and Water Intake

The food and water intake was not affected in the control group. It was increased by only 30% in the nonpregnant group exposed to the LPHC diet from days 1 and 2 (Figure 1(a)).

#### 3.1.1. Control Group

The control group was characterized by fluctuations in food and water intake in nonpregnancy and pregnancy. These fluctuations in food intake were similar in the control group and the LPHC group (Figures 1(a) and 1(b)). Regarding body weight, we observed an increase trend of approximately 20%, compared to the LPHC group when the pregnancy started, but in the following 20 days, throughout pregnancy, we observed weight gain of 25% (Figure 2(b)). The control diet nonpregnant (CNP) group rats consumed 45 to 80 kcal/d of food and 20-60 ml/d of water. The pregnant rats fed with the control diet (CP) consumed 40 to 118 kcal/d, showing a tendency to increase at days 11, 17, 19, and 20G (110 and 115 kcal/d). This group showed a water intake of 35-60 ml/d, showing a reduction at day 14G (70 ml/d) (Figures 1(a)–1(d)).

#### 3.1.2. LPHC Group

The LPHC nonpregnant (NPLPHC) group consumed 80 to 90 kcal/d of food (Figure 1(a)) and 20-80 ml/d of water (Figures 1(a) and 1(c)) and presented a weight gain of 5% (Figures 2(a) and 2(c)). The LPHCP group consumed 45 to 118 kcal/d of food (Figure 1(b)), which increased on days 10, 12, 13, and 14G (110-118 kcal/d), and 20-50 ml/d of water (Figure 1(d)), which decreased on day 15G (15 ml/d) and showed a weight gain of 20% (Figure 2(b)). In Figure 1, we show the temporal pattern of food and water intake (Figures 1(b) and 1(d)), as well as body weight (Figure 2(b)).

Rats with the LPHC diet showed a very similar pattern in food and water intake (Figure 1(c)).

Indeed, because of the diet composition, we fed the control rats with a larger proportion of carbohydrates throughout the protocol (Figure 1(d)). We observed a 60% increase in food consumption in the nonpregnant rats that consumed the LPHC on days 1 and 2 (20 ± 1 vs. 12 ± 0.7 g/d (80 ± 4 vs. 48 ± 2.8 kcal/d) (*p* < 0.05). On subsequent days of exposure, no significant changes were observed; food consumption ranged from 20 to 22 g/d, corresponding to 80 to 82 kcal/d.

In the nonpregnant group, no differences were observed in food intake except on days 1 and 2 of exposure to the diet, in which we observed a 40% decrease in the control group. In this same group, we observed a greater fluctuation in water intake in the control diet compared with the LPHC diet, and an increase of 20% was observed on days 3 and 4, with a decrease of 80% on days 5, 6, and 7 in the control group compared with LPHC. During pregnancy, no differences in food intake in both groups were found; the behavior throughout the pregnancy was similar in both groups. The same occurred with water intake, which decreased between days 13 and 14 of pregnancy.

### 3.2. Weight Gain

Figures 2(a) and 2(b) show that, in nonpregnant rats, the weight gain was similar in both groups: control and LPHC; in early pregnancy, weight gain increased by 20 to 30% in the control and LPHC groups, respectively, while in late pregnancy, an increase of approximately 40 to 45% was observed in both groups.

### 3.3. Energy Metabolism

#### 3.3.1. Food Intake

Figure 3 shows the daily parameters obtained in metabolic cages during 24 h, with lights on at 07:00 h and off at 19:00 h. Nonpregnant rats showed an increased food intake (Figure 3(a)) observed at 11:00 h in the control group when compared to the LPHC group (4.00 ± 0.01 vs. 2.00 ± 0.01 g) (*p* < 0.05).

Nonpregnant rats within the control group showed food intake ranging from zero to 4 g during the light period, while the experimental group food intake ranged from zero to 2 g. During the dark period, the food consumption was similar (from 8 to 12 g in both groups) in early and late pregnancy (Figures 3(b) and 3(c)).

In early pregnancy (pregnant 8G), the rats exposed to the control diet during the light period showed a food intake that ranged from zero to 7 g, while it ranged from 4 g to 13 g in the LPHC group (Figure 3(b)). In the dark period, food intake was 8 g to 20 g in the control group and 13 g to 25 g in the LPHC group (Figure 3(b)).

In late pregnancy (15G), the rats with the control and experimental diet showed a food intake that ranged from zero to 8 g during the light period, and food intake ranged from 8 g to 22 g during the dark period; no significant differences were found (Figure 3(c)).

### 3.4. Energy Expenditure

#### 3.4.1. Nonpregnant Rats

In nonpregnant rats within the control group, we observed a significant decrease in energy expenditure (EE) at hours 3 and 4 during the light period, when compared to the LPHC group (52 ± 0.01 vs. 57 ± 0.01 kcal) (*p* < 0.05). During the light period, in nonpregnant rats within the control group, we observed energy expenditure that ranged from 52 to 80 kcal, while in the LPHC group, it ranged from 62 to 80 kcal. During the night period, the energy expenditure ranged from 82 to 62 kcal in both groups. We observed an increase in EE in nonpregnant rats exposed to the LPHC diet at hours 15, 16, and 17 during the dark period, when compared to the control group (80 ± 0.01 vs. 78 ± 0.01 kcal, hour 15) (77 ± 0.01 vs. 72 ± 0.01 kcal, hour 16) (82 ± 0.01 vs. 70 ± 0.01 kcal, hour 17) (*p* < 0.005) (Figure 3(d)).

#### 3.4.2. Pregnant rats

In contrast to nonpregnant rats, in pregnant rats, we did not observe significant differences on EE in both stages, early and late pregnancy (Figures 3(e) and 3(f)), during either light or dark periods. In the dark period, the EE ranged from 80 to 90 kcal in the control group and from 70 to 80 kcal in the LPHC group. In late pregnancy, we observed a decrease in EE on hour 2 of the light period in the LPHC group (52 ± 0.01 vs. 63 ± 0.01 kcal), when compared with the control group.

### 3.5. Respiratory Quotient (RQ)

#### 3.5.1. Nonpregnant Rats

In nonpregnant rats with the control diet (Figure 4(a)), we observed an increase in the RQ rhythmicity during daytime at hours 4, 5, 7, 8, and 9 (the value ranged from 0.8 to 1) (*p* < 0.05), when compared with the LPHC group. This finding indicates that, during the light period, carbohydrates are metabolically oxidizing in the control group, while in the LPHC group, lipids were oxidized (Figure 4(d)). During the dark period, the RQ showed a significant increase in the control group at hours 22, 23, and 24, when compared to the rhythmicity in the LPHC group (the value ranged from 1 to 1.1), indicating carbohydrate oxidation, with a decrease at hours 18, 19, and 20 (Figure 4(e)).

During the dark period, there was a decrease in the rhythmicity of RQ in the nonpregnant rats with the LPHC diet compared to the nonpregnant rats exposed to the control diet (Figure 4(e)).

#### 3.5.2. Pregnant Rats

Pregnant rats within the control group showed a decrease in RQ rhythmicity during the dark period. This finding suggests lipid oxidation in the control group, in both the light and dark periods. The LPHC group showed a differential energy metabolism between nonpregnant and pregnant rats. The LPHC group showed a diurnal oxidation shifted away from lipids in favor of carbohydrates (Figure 4(b)). In pregnant rats in the early pregnancy period, the LPHC group showed a significant increase in the diurnal rhythmicity of the RQ at hour 9 (the value was 0.9) (*p* < 0.05), when compared to the control group (Figure 4(b)). During the dark period, we observed an increase in the RQ rhythmicity in the LPHC group at hours 15, 16, 17, and 18, when compared to the control group (the value ranged from 1 to 1.03), indicating carbohydrate oxidation.

When representing the area under the rhythmicity curve, the RQ showed a decrease during the light period in pregnant rats exposed to the control diet on day 8G, when compared to the LPHC group (Figure 4(d)).

The intragroup comparisons showed an increase in RQ rhythmicity on day 15G in the control group, when compared to day 8G pregnant rats exposed to the control diet (Figures 4(f) and 4(i)). These comparisons also showed an increase in RQ rhythmicity on day 15G in the LPHC group, when compared to nonpregnant rats that consumed the control diet (Figure 4(i)).

We did not observe any significant difference in the RQ rhythmicity in the late pregnancy stage during the light period (Figure 4(c)). However, in the dark period, we observed an increase in the RQ rhythmicity in the LPHC group at hours 12 and 13 (the value ranged from one to 1.03) (Figure 4(c)), indicating carbohydrate oxidation. In the dark period, no oscillations showed. The oscillations are considered in periods of 24 h and light vs. dark in both the control group and the LPHC group.

### 3.6. Visceral Fat Concentration

#### 3.6.1. Nonpregnant Rats

In the nonpregnant rats with the control diet, we observed an increase in VAT concentration, when compared to the LPHC group (2.00 ± 0.07 vs. 8.80 ± 0.07 g).

#### 3.6.2. Pregnant Rats

In contrast, in pregnant rats, the VAT concentration decreased in the LPHC group, compared to the nonpregnant rats. Intragroup comparisons in the group exposed to the LPHC diet showed an increase of 600 and 610% on days 8G and 15G (6.00 ± 2.00 vs. 1.00 ± 0.03 g and 6.20 ± 20 vs. 1.00 ± 0.03 g, respectively), when compared to the nonpregnant group with the LPHC diet. In addition, we observed an increase of 200% in pregnant rats on day 15G, compared to the pregnant rats on day 3G (6 ± 2 vs. 2 ± 0.03 g).  Figure 5(a) shows the visceral fat concentration in nonpregnant and pregnant female rats in both the early and the late pregnancy period.

### 3.7. Free Fatty Acid Levels

#### 3.7.1. Nonpregnant Rats

In nonpregnant rats in the control group, we observed a 333% increase, when compared to nonpregnant female rats exposed to the LPHC diet (0.60 ± 0.20 vs. 0.18 ± 0.02 nmol/*μ*l) (*p* < 0.05) (Figure 5(b)).

#### 3.7.2. Pregnant Rats

Pregnant rats consuming the control diet showed a 200% decrease on day 8G and a 130% decrease on day 15G, both when compared to the nonpregnant rats.

Comparing pregnant rats to nonpregnant rats with the LPHC diet, we observed a 50% decrease on day 8G (0.20 ± 0.01 vs. 0.40 ± 0.02 nmol/*μ*l) (*p* < 0.05) and a 43% decrease on day 15G (0.23 ± 0.01 vs. 0.40 ± 0.02 nmol/*μ*l) (*p* < 0.05). Figure 5(b) shows fatty acid levels in serum in nonpregnant and pregnant rats.

### 3.8. Biochemical Markers in Serum

#### 3.8.1. Triacylglycerides

TG levels in serum increased on day 8G in the control group compared to the LPHC group. Intragroup comparisons showed an increase in TG levels in serum on day 15G in the control group, compared to nonpregnant rats. In pregnant rats, the TG levels in serum increased on day 15G in the LPHC group, compared to the nonpregnant rats (210 ± 25 vs. 100 ± 5 mg/dl) (*p* < 0.05) (Figure 6(a)).

#### 3.8.2. Cholesterol

Cholesterol levels in serum decreased on day 15G in pregnant rats exposed to the LPHC diet, compared to the nonpregnant rats exposed to the same diet. Intragroup comparisons showed a decrease in cholesterol levels in serum on day 15G in the control group, compared to the nonpregnant rats, and we observed a similar behavior in the LPHC group on day 8G LPHC (Figure 6(b)) (60 ± 0.2 vs. 110 ± 0.4 mg/dl) (*p* < 0.05).

#### 3.8.3. High-Density Lipoproteins (HDL)

No significant differences were found in the concentration of HDL in serum; however, we observed a tendency to decrease on late pregnancy (15G). A decrease in HDL levels in serum was observed in pregnant rats that were exposed to the LPHC diet on day 15G, when compared to nonpregnant rats exposed to the same diet (39 ± 1.5 vs. 48 ± 2 mg/dl) (*p* < 0.05). This behavior was similar in both the control group and the LPHC group (Figure 6(c)).

#### 3.8.4. Glucose

We observed a decrease in glucose levels in serum on day 15G in the control group in pregnant rats, compared to nonpregnant rats. This behavior was similar in the LPHC group (Figure 6(d)).

Increase in serum glucose concentration was shown in pregnant rats that consumed the LPHC on 8G when compared to nonpregnant rats that consumed the LPHC (170 ± 3 vs. 140 ± 2 mg/dl) (*p* < 0.05); also, a decrease in serum glucose concentration was shown in pregnant rats that consumed the LPHC on 15G when compared to pregnant rats on day 8 that consumed the LPHC (110 ± 2 vs. 170 ± 3 mg/dl) (*p* < 0.05).

#### 3.8.5. Insulin

In contrast, in the LPHC group, we observed a tendency to increase on day 15G, compared to the nonpregnant rats on day 8G. Decrease in insulin concentration in serum was observed in pregnant rats in LPHC on day 8G when compared to pregnant rats with the control diet (5 ± 2 vs. 12 ± 2 *μ*lU/ml) (*p* < 0.05). On 15G, increase was observed in rats with LPHC compared to the pregnant rats with the control diet (13.8 ± 3 vs. 5 ± 2 *μ*lU/mL) (*p* < 0.05) (Figure 6(e)).

Figure 6 shows the concentration of triacylglycerides, cholesterol, high-density lipoproteins, glucose, and insulin in serum.

## 4. Discussion

Pregnancy is accompanied by important changes in the maternal metabolism [13]. In normal conditions, early gestation implies an anabolic state, where an increased fat storage and unchanged insulin sensitivity occur. In contrast, late gestation implies a catabolic state, where decreased insulin sensitivity occurs, along with an increased maternal blood glucose and FFA levels, in order to fulfill the demands of the growing fetus [14]. In this research, we studied the effects of the LPHC diet on RQ and VAT during early and late pregnancy. We studied two metabolic challenges, a nutritional one (related to the LPHC intake) and a psychological one (related to the condition of pregnancy). Within this framework, pregnancy requires more energy, and the LPHC diet has a limited source of amino acids, which leads to a reduction in the biochemical capacities of the system. Our longitudinal study showed that food intake in rats was similar during pregnancy in both groups of rats: the one exposed to the LPHC diet and that exposed to the control diet. This result is consistent with other reports that have showed that food intake during pregnancy tends to compensate for deficient nutrient availability to achieve fetal development [15]. Other reports have indicated that diet composition is a contributing factor in the process of feeding [16]. On its part, water intake in nonpregnant rats exposed to the LPHC diet may be due to a physiological response to protein restriction. Nonpregnant rats exposed to the LPHC diet showed a decrease in food consumption at hour 11 of the light period, which could be due to a response to the change in the light-dark cycle [17]. Pregnant rats during both early and late pregnancy showed no changes in food intake during either light or dark periods. In the gestation period, food intake tends to be regulated when facing nutritional restrictions [18].

### 4.1. Energetic Metabolism

Knowing the changes in RQ during pregnancy allows a better understanding of the anabolic/catabolic states. The anabolic state is characterized by an increase in RQ, which is associated with fat storage, as well as a decreased endogenous fat oxidation while the catabolic state is rather characterized by a decrease in RQ, which is due to a greater demand for energy during pregnancy, not only associated with an increase in total food intake but also due to the mobilization and increase in fat [11].

The pregnant rats exhibit metabolic and hormonal adaptations *per se*, which are expressed in energetic adaptations as the gestation progresses. In the present study, nonpregnant rats did not show changes in food consumption during the daytime cycle, in either group (control and experimental). This indicates that rats were in a state of absolute rest. In early pregnancy, during the daytime cycle, the rats exposed to the LPHC diet showed a tendency to increase food intake, which ranged from 7 to 12 g/h. In late pregnancy, in this same light period, the rats also showed a tendency to increase food intake in both groups exposed to the LPHC diet. This tendency increased starting at hour 6, by a quantity that ranged from 4 to 10 g/h. In early pregnancy, in the dark period, rats exposed to the LPHC diet showed a tendency to increase food intake. Early gestation implies an anabolic state that leads to increased fat storage and mostly unchanged insulin sensitivity [14] in normal conditions; however, when the diet has a restriction of protein, it could modify the regulation of food intake.

In relation to RQ, in nonpregnant rats exposed to the control diet, we observed an increase in this indicator during the dark period, which suggests a greater lipid oxidation. In the light period, a tendency to increase was only observed in the control group. The RQ is an indirect index of the nature and proportion of substrates utilized to meet energy needs [19]. In early pregnancy, during the light period, the RQ value increased in the group exposed to the LPHC diet, indicating more carbohydrate oxidation. In the dark period, no changes in the RQ were shown, when compared to the control group, indicating that the metabolism responds differently to the intake of the LPHC diet during pregnancy in the light and dark period. This variation in the RQ suggests that the oxidation of lipids and carbohydrates is an adjustment to face protein requirements. In late pregnancy, we observe an increase in the RQ in the group exposed to the LPHC diet during the light period, indicating more carbohydrate oxidation. In the night period, no changes were observed; in both groups, the RQ was in the range of 1.0 or greater than 1, which indicates exclusive carbohydrate oxidation. Intergroup comparisons showed an increase in the RQ in rats exposed to the control diet in late pregnancy, when compared to early pregnancy. This suggests that, in early pregnancy with the control diet, the lipids are predominantly oxidized, and carbohydrates are oxidized in late pregnancy, confirming that the experimental diet contains higher carbohydrates and lower protein content compared to the control diet.

The results observed on energy expenditure suggest and confirm that, during this rheostatic state, the maternal environment tends to regulate energy consumption and expenditure [8].

The RQ increased during pregnancy compared to a nonpregnant state; other studies showed that RQ values tended to vary more among nonpregnant than pregnant rats [11].

In this study, intergroup comparisons showed an increase in the RQ in rats fed with the LPHC diet in late pregnancy, when compared with those in the early pregnancy stage. This suggests that carbohydrates were oxidized in both early and late pregnancy with the LPHC diet. It has been suggested that prenatal protein restriction increases the risk of low body weight in the offspring through mechanisms that affect thermogenesis [20]. This indicator tends to increase in both periods (light and dark) in rats exposed to the LPHC diet, when it is recorded in the metabolic cages for 24 h a day, and it did not affect EE in the maternal environment. As reported in several studies (in particular the prospective ones), the RQ was moderately higher during pregnancy compared to the nonpregnant state [11].

During the light period, the rats' sleep time as well as the oxidation of fatty acids decreases, favoring the oxidation of glucose, which correlates with serum free of fatty acids and with the increase in visceral adipose tissue. Conversely, we found a decrease in VA in nonpregnant rats exposed to the LPHC diet, which suggests an activation of a differential energy metabolism in response to the diet. It is known that during pregnancy, the main fuel in the maternal environment is carbohydrates, and energy demand is greater during early pregnancy given the development of the new organism and the changes that the maternal tissues undergo [21, 22]. Lipid metabolism during gestation, as well as glucose and protein metabolism, was modified. These results suggest that this behavior of fat is due to an effect of pregnancy in response to the consumption of the LPHC diet. The increase in carbohydrate intake compensated by a decrease in fat intake during the course of pregnancy would also increase the RQ, corresponding to the RQ of the diet [11].

### 4.2. Visceral Fat Concentration

In the present study, an increase in fat was observed in pregnant rats exposed to the LPHC diet in both early and late gestation. However, no changes in body weight were observed, which could be related to a physiological adaptation to protein restriction in the diet during pregnancy [23, 24]; the opposite occurs in nonpregnant rats. The fat accumulation in the mother is required for rapid growth of the fetal-placental unit during the second half of the pregnancy [11, 25], probably to prevent or minimize the effect of marginal food intake on the fetus by the mother. Under normal conditions, protein requirements are known to be needed for structural purposes, as well as carbohydrates as a fuel source [26–28].

The increase in insulin in serum, a lipogenic hormone, in early pregnancy seems to produce an environment that favors maternal fat accumulation. Fat accumulation in the mother is required for the rapid growth of the fetal-placental unit during the second half of pregnancy [25], probably to prevent or minimize the effect of the mother's deficient food intake on the fetus. Moreover, increased levels of hormones such as oestrogen, progestins, and cortisol in early pregnancy stimulate fat accumulation as well.

### 4.3. Biochemical Markers

Changes in lipid and glucose metabolism in the maternal environment are considered necessary for fetal development [29, 30]. Previous studies show that high triacylglyceride levels in early gestation are related to birth weight and fetal macrosomia [31]. In this study, we observe an increase in triacylglyceride levels in serum increase (110%) as well as decreases in cholesterol and glucose levels (45 and 26%, respectively) in the late pregnancy stage with the LPHC diet, in comparison to nonpregnant rats. This could be a response to the consumption of the LPHC diet during pregnancy. Assessing the RQ in pregnancy is not only of physiological relevance but also of nutritional interest; the observed changes in serum markers suggest an adaptation during pregnancy, in which there are an increase in the levels of triacylglycerides and decreases in serum cholesterol and glucose, to favor the development of the new organism.

In conclusion, consuming a LPHC diet during early pregnancy increases the RQ, VAT, and fatty acid levels in maternal environment, suggesting that LPHC intake increases the glucose metabolism as a compensatory mechanism to meet energy needs of the fetus and the mother during pregnancy.

## Data Availability

The authors confirm that the data supporting the findings of this study are available.
